# Book Reviews

**Published:** 1896-02

**Authors:** 


MEDICAL DIAGNOSIS WITH SPECIAL REFERENCE TO PRACTICAL MEDICINE. A Guide to the Knowledge and Discrimination of Diseases. By J. M. DACOSTA, M. D., LL. D., President of the College of Physicians of Philadelphia ; Emeritus Professor of Practice of Medicine and of Clinical Medicine at the Jefferson Medical College, Philadelphia ; Physician to the Pennsylvania Hospital, etc. Octavo, pp. 1104. Illustrated with engravings on wood. Eighth edition, revised. Philadelphia : J. B. Lippincott Company. 1895.

This treatise on medical diagnosis has been before the profession over thirty years and has now passed through eight editions —an average of one every four years. It has been used as a textbook in nearly every medical college in this country since its first appearance in 1864, hence is well known to teachers, practisers and students of medicine. It is as nearly a medical classic as any contemporaneous medical literature can ever become.

Though the progress of medicine has been marvelous since DaCosta's first edition was published, during which period methods of diagnosis have been ever changing and advancing toward perfection, yet the author has kept his treatise always abreast of the period, and it is still a trustworthy exponent of the most valuable modern thought in the field of medical diagnosis.

To undertake a systematic review of a treatise so widely and favorably known would be almost a thankless task, since we could hardly expect our readers to give us their patient hearing during such a discourse; but we beg their attention long enough to notice the changes in the new edition. The author has revised the work and altered some of its chapters; he has introduced new matter and has condensed some portions of the old; while he has endeavored to incorporate such bacteriological facts as appear to have been established as valuable for diagnostic purposes. Finally, a number of new wood-cuts have been introduced as well as some temperature charts taken from personally observed cases, all of which contribute to elaborate the text. We think, however, there is still much room for the employment of the engraver's art toward the improvement of this treatise. Da Costa's medical diagnosis will long remain a much-sought book in the libraries of progressive physicians.

Supplement to the International Encyclopedia of Surgery. Edited by John Ashhurst, Jr., M. D., LL. D., Philadelphia. One royal 8vo volume of 1,136 pages, illustrated by numerous wood-engravings and a chromo-lithographic plate. Cloth, $7.50 ; leather, $8.50. To subscribers to the entire set, cloth, $6 ; leather, $7, and half-morocco, $8. New York : William Wood & Co. 1895.

The *International Encyclopedia of Surgery* commenced its appearance in 1881, when its first volume bears date, and it is now seven years since the last volume of the series was published. The book consists of fifty-five separate articles, written by as many different authors. The object of this supplementary volume is to fill the gap that has occurred in surgical literature since that time. How satisfactorily this has been done by the issuance of this volume is not easily determined. The aim has been to bring forward each of the original subjects to the present period, thus practically modernising the encyclopedia in order to make it a working guide for the surgeon of today. In some of these branches there has been great change in methods of practice ; in others but few, if any, hence the articles are variable in length. One of the most important chapters relates to cerebral surgery and is written by W. W. Keen, and another possessing the keenest interest relates to injuries and diseases of the abdomen by Albert Vander Veer. Another important chapter, the last in the book, relates to the construction and organisation of hospitals by Edward Cowles.

The mechanical execution of the book is all that could be desired and its price is moderate. It is sold by subscription only, hence orders should be placed with the publishers direct or through their authorised agents. To those who possess the preceding volumes this one becomes almost a necessity.

AN AMERICAN TEXT-BOOK OF OBSTETRICS FOR PRACTITIONERS AND STUDENTS. RICHARD C. NORRIS, M. D., Editor, Robert L. Dickinson, Art Editor. Royal 8vo, pp. 1009. With nearly 900 colored and half-tone illustrations. Price, $7.00, cloth; $8.00, sheep; $9.00, half-Russia. Philadelphia: W. B. Saunders, 925 Walnut street. 1895.

It is announced that this work owes its existence to the fact that it seemed practicable to produce a work that should not only embody the teachings of several prominent obstetricians, but should also be a standard teaching work for students and a guide for practitioners. The authors then further announce that they have selected those teachers of obstetrics in several of the leading schools and hospitals of America that possess experience. While this may be true as an abstract fact, we think it will strike many readers of the book, especially those of the widest knowledge of the literature of obstetrics, that some of the authors have been oddly chosen. There are many teachers in the schools of America that are, at least, more experienced and certainly better suited to authorship than some whose names appear on the title page of this treatise.

While there is much opportunity for just criticism of this work, there is yet much in it that merits praise. First of all, the reader is impressed with the excellence of the plates and drawings. Probably no obstetric treatise, certainly none in America, has ever presented such a wealth of artistic illustration as does this one. Moreover, the pictures are, for the most part, anatomically, physiologically and pathologically correct. Too much praise cannot be given to the art editor, Dr. Dickinson, who we understand is willing to be held responsible for the illustrations in all departments.

We do not intend to give an analytical review *seriatim* of this treatise, but simply to speak of features here and there that impress us either as worthy of praise or blame. Next, we may speak of the first and second sections relating to the anatomy of the generative organs and the physiology of pregnancy, both written by George A. Piersol. It is doubtful if these two subjects have ever, heretofore, been as well presented—certainly not in a work on obstetrics. A careful study of these two subjects as here presented ought to afford, even the novitiate, a pretty complete knowledge of them. Nay, more; we may affirm that after such a study of Piersol's presentation of the physiology of pregnancy, there can be no excuse on the part of the student for an imperfect or lax knowledge of the subject. The physiology of labor is another section that will attract the reader as specially deserving of commendation. It is a clear, concise and well-illustrated setting forth of the subject and is written by Robert L. Dickinson. Next, we would commend the section on the mechanism of labor, by Edward Reynolds. Rarely, if ever, has this subject, one so difficult for the student to understand, been treated so clearly, elaborately or adequately. Finally, the section on obstetric surgery, divided into three parts, instrumental operations by Cameron, manual operations by Dickinson, and celiotomy for sepsis in the child-bearing period by Hirst, invites attention quite as much for its shortcomings as for its merits. In a work of the pretensions of this one it would seem appropriate to invite the most experienced obstetric operators, those of the largest surgical knowledge, to contribute the material for the section on obstetric operations. It should be made so exhaustive as to leave nothing to be desired. In the present instance, while much is commendable, there is yet an incompleteness that challenges remark. This is especially true of the final subdivision. In the first place, the execrable word celiotomy should be expunged from medical literature ; it certainly has no place in the present treatise, which should aim to give the best of everything, even in nomenclature. There is one beautiful plate in this section showing diphtheritic endometritis of the puerperal womb, but the section itself can hardly be said to be authoritative on the subject.

It is difficult, it must be admitted, in a work of this kind, to avoid repetition or prevent conflict of opinion, but the editors in this instance have succeeded in reducing these perplexities to a minimum. It has been doubted in some quarters whether another work on obstetrics is needed at this time, and again it has been questioned whether obstetrics is a proper subject for a syndicate of authors to write upon. We do not propose to discuss either of these questions in connection with this book. It is a work of much merit and will easily take its place alongside of the other volumes of the American text-book series ; indeed, deserving a place at the head of the list.

